# ChatGPT in Oral Pathology: Bright Promise or Diagnostic Mirage

**DOI:** 10.3390/medicina61101744

**Published:** 2025-09-25

**Authors:** Ana Suárez, Yolanda Freire, Víctor Díaz-Flores García, Andrea Santamaría Laorden, Jaime Orejas Pérez, María Suárez Ajuria, Juan Algar, Carmen Martín Carreras-Presas

**Affiliations:** 1 Department of Pre-Clinic Dentistry II, Faculty of Biomedical and Health Sciences, Universidad Europea de Madrid, 28670 Madrid, Spain; ana.suarez@universidadeuropea.es (A.S.); yolanda.freire@universidadeuropea.es (Y.F.); andrea.santamaria@universidadeuropea.es (A.S.L.); jaime.orejas@universidadeuropea.es (J.O.P.); mariaana.suarez@universidadeuropea.es (M.S.A.); 2 Department of Pre-Clinic Dentistry I, Faculty of Biomedical and Health Sciences, Universidad Europea de Madrid, 28670 Madrid, Spain; 3 Department of Clinic Dentistry, Faculty of Biomedical and Health Sciences, Universidad Europea de Madrid, 28670 Madrid, Spain; juan.algar2@universidadeuropea.es; 4 Department of Clinical Dentistry-Postgraduate Studies, Faculty of Biomedical and Health Sciences, Universidad Europea de Madrid, 28670 Madrid, Spain; carmen.martin2@universidadeuropea.es

**Keywords:** ChatGPT, oral pathology, oral squamous cell carcinoma (OSCC), oral leukoplakia (OL), oral lichen planus (OLP), diagnostic accuracy, multimodal large language models (LLMs)

## Abstract

*Background and Objectives:* The growing academic interest within the biomedical sciences regarding the diagnostic capabilities of multimodal language models, such as ChatGPT-4o, is clear. However, their ability to interpret oral clinical images remains insufficiently explored. This exploratory pilot study aimed to provide preliminary observations about the diagnostic validity of ChatGPT-4o in identifying oral squamous cell carcinoma (OSCC), oral leukoplakia (OL), and oral lichen planus (OLP) using only clinical photographs, without the inclusion of additional clinical data. *Materials and Methods:* Two general dentists selected 23 images of oral lesions suspected to be OSCC, OL, or OLP. ChatGPT-4o was asked to provide a probable diagnosis for each image on 30 occasions, generating a total of 690 responses. The responses were then evaluated against the reference diagnosis set up by an expert to calculate sensitivity, specificity, predictive values, and the area under the ROC curve. *Results:* ChatGPT-4o demonstrated high specificity across the three conditions (97.1% for OSCC, 100% for OL, and 96.1% for OLP), correctly classifying 90% of OSCC cases (AUC = 0.81). However, this overall accuracy was largely driven by correct negative classifications, while the clinically relevant sensitivity for OSCC was only 65%. In spite of that, sensitivity was highly variable: 60% for OL and just 25% for OLP, which limits its usefulness in a clinical setting for ruling out these conditions. The model achieved positive predictive values of 86.7% for OSCC and 100% for OL. Given the small dataset, these findings should be interpreted only as preliminary evidence. *Conclusions:* ChatGPT-4o demonstrates potential as a complementary tool for the screening of OSCC in clinical oral images. Nevertheless, the pilot nature of this study and the reduced sample size highlight that larger, adequately powered studies (with several hundred cases per pathology) are needed to obtain robust and generalizable results. Nevertheless, its sensitivity remains insufficient, as a significant proportion of true cases were missed, underscoring that the model cannot be relied upon as a standalone diagnostic tool.

## 1. Introduction

Artificial intelligence (AI) tools based on language models have emerged as potential support systems in biomedical and dental practice [1,2]. These models are trained on extensive textual datasets. They can interpret clinical information, generate diagnostic hypotheses, and provide reasoned justifications for their responses [3,4]. This makes them valuable aids in clinical decision-making.

Beyond textual analysis, some of these systems have demonstrated remarkable abilities to interpret descriptions of clinical images, identify patterns indicative of disease and support diagnostic reasoning. This has been demonstrated even when the systems were not specifically trained on medical data [5,6,7,8]. In dentistry, where many pathologies manifest as signs or lesions in the oral cavity [9], the potential of these models is especially promising.

Their ability to interpret clinical images indirectly, through descriptions or categorizations, and to reason about possible diagnoses could contribute to the early detection of potentially malignant oral disorders (OPMDs) [10,11,12]. These emerging applications could provide novel support tools to complement traditional clinical evaluation, help screening in areas where specialists are scarce, and enhance patient safety through automated second opinions [12,13,14].

Early detection of oral squamous cell carcinoma (OSCC), oral leukoplakia (OL) and oral lichen planus (OLP) is critical to improving patient prognosis and avoiding complications associated with a delayed diagnosis [15,16,17,18,19,20]. However, these lesions can present with variable clinical appearances and are often underdiagnosed or mistaken for benign conditions, delaying their recognition [21,22,23,24,25]. Consequently, there is a growing interest in exploring digital tools that can help healthcare professionals evaluate these pathologies initially, especially in settings with limited resources.

ChatGPT, a large language model (LLM) developed by OpenAI (San Francisco, CA, USA), has demonstrated emerging capabilities in text generation, semantic understanding, and answering clinical questions [26,27,28,29]. Although ChatGPT was not specifically designed for diagnostic purposes, its use in clinical simulations, decision-making processes, and educational contexts has opened new avenues of research into its potential applications in healthcare [26,30,31].

Unlike traditional LLMs, which focus on natural language, more recent multimodal models such as ChatGPT-4o also analyze visual information [32]. Thanks to their transformer-based architecture, these models can simultaneously integrate textual and image data, enabling them to interpret visual content with a level of accuracy that rivals human performance in certain cases [33].

Although deep learning approaches, such as convolutional neural networks (CNNs), have been specifically trained on large datasets of labeled oral images and have demonstrated a high level of sensitivity and accuracy in the detection of oral cancer and potentially malignant disorders, the same cannot be said for general-purpose, multimodal large language models (LLMs). Unlike CNNs, which are optimized for image recognition tasks, LLMs such as ChatGPT-4o were not originally designed to analyze medical photographs. Nevertheless, their ability to process both text and images has opened up new avenues for research in clinical decision support [10,12,13]. Despite this potential, their diagnostic behavior when analyzing clinical photographs unaided remains under-explored. In dentistry, this scenario is particularly relevant since many conditions manifest primarily as visible mucosal changes that can be documented with simple photographs. This makes the image-only context both common in practice and clinically significant.

To address this gap, a constrained first-impression scenario was designed in which ChatGPT-4o is provided with only a clinical photograph and must return a single probable diagnosis without access to additional clinical context or step-by-step guidance. We quantify core diagnostic indicators for three common conditions: oral squamous cell carcinoma (OSCC), oral leukoplakia (OL) and oral lichen planus (OLP), including sensitivity, specificity, predictive values and the area under the ROC curve. By making the prompting protocol, repetition scheme, and analysis plan explicit, this exploratory pilot study provides transparent and reproducible measurements, as well as practical boundaries for future clinical and technical research.

In this context, the present study preliminarily explores ChatGPT-4o’s ability to recognize OSCC, OL, and OLP from selected clinical images. The study aims to provide initial evidence of the model’s diagnostic behavior in a simulated environment.

## 2. Materials and Methods

### 2.1. Ethical Considerations

This study was conducted as a retrospective exploratory analysis for academic purposes. The images used were obtained from a teaching database that had been authorized for educational and research activities. The images contained no patient-identifying information, and their use was approved through written informed consent, which was obtained after the participants had been properly informed.

The research was approved by the Ethics Committee of the European University of Madrid (approval code 2025-56) and was conducted in accordance with the ethical principles of the Declaration of Helsinki. To preserve confidentiality, all interactions with the model were performed using ChatGPT’s temporary chat function, which prevents the permanent storage of conversations [34].

Data processing was conducted in compliance with Regulation (EU) 2016/679 of the European Parliament and of the Council on the protection of personal data.

### 2.2. Identification of Clinical Images

Two general dentists, who had not received specific training in oral pathology and had no access to additional diagnostic information, reviewed a set of 30 clinical images of oral lesions obtained from an oral pathology specialist’s private repository. Their goal was to select images that appeared to be oral squamous cell carcinoma (OSCC), oral leukoplakia (OL), or oral lichen planus (OLP). They could assign one or more of these conditions to each image. Importantly, this step did not determine the ground truth, which was established independently by an oral pathology specialist.

### 2.3. Generation of Responses with ChatGPT

For each suspicious image, ChatGPT-4o (OpenAI, San Francisco, CA, USA) was given the following prompt: “As an expert in diagnosing oral lesions, please analyze the provided photograph and suggest a probable diagnosis”, without providing any additional clinical context.

This simple formulation was deliberately selected to simulate a “first-impression” diagnostic scenario, in which the model was asked for a probable diagnosis without guidance or structured input.

Thirty independent responses were generated for each image, with a new conversation initiated each time to avoid bias and reflect the model’s probabilistic behavior. These repetitions were conducted over a two-week period, at different times of the day, to capture potential temporal variability in the model’s outputs. The premium version of ChatGPT-4o (subscription-based) was used to ensure stable access to multimodal capabilities. Interactions were carried out at various times of the day using the “temporary chat” function to prevent the storage of conversations. All responses were recorded in Microsoft Excel (Microsoft, Redmond, WA, USA).

### 2.4. Establishment of the Gold Standard

The images selected by the general dentists were evaluated by an oral pathology specialist, who made the final diagnosis of OSCC, OL, or OLP based on clinicopathological correlation, including histopathological confirmation when available. The expert was blinded to the classifications made by the general dentists and by ChatGPT, ensuring the independence of the reference standard. Therefore, the gold standard was not affected by the first selection process, which merely simulated a real-world triage by non-specialists.

This expert judgment was considered the gold standard for evaluating the diagnostic validity of the model’s results.

### 2.5. Data Analysis

A 2 × 2 contingency table was constructed for each clinical entity (OSCC, OL, and OLP), comparing ChatGPT’s responses with the gold standard. The main diagnostic validity indicators were then calculated from these tables: sensitivity, specificity, positive predictive value (PPV), negative predictive value (NPV), false positive rate, false negative rate, proportion of correctly classified cases, and area under the ROC curve (AUC).

All results were reported with their corresponding 95% confidence intervals. Statistical analysis was performed using Stata software (version BE 14, StataCorp, College Station, TX, USA).

## 3. Results

The general dentists found a total of nine images as suspicious for OSCC, four as possible OL, and ten as suggestive of OLP. Following specialist evaluation, OSCC was confirmed in two of the nine initially selected images, OL in three of the four, and OLP in four of the ten considered suggestive.

Table 1 shows the diagnoses issued by ChatGPT for each of the identified images across the 30 repetitions. Table 2 presents the diagnostic validity measures of ChatGPT for the three pathological entities of interest.

## 4. Discussion

In this study, ChatGPT-4o demonstrated an elevated level of specificity (97.14%) and achieved an overall classification accuracy of 90% in the diagnosis of OSCC, with an area under the ROC curve exceeding 0.8.

It is important to stress, however, that this clear high accuracy was primarily due to the considerable number of correctly found negative cases. The sensitivity for OSCC was only 65%, meaning that a substantial fraction of true positive cases was overlooked. From a clinical standpoint, sensitivity is the more meaningful metric, as missing malignant lesions can have profound consequences. This limitation restricts the applicability of ChatGPT-4o in real diagnostic contexts, despite its encouraging specificity.

Similar findings have been reported in a recent study, where GPT-4 achieved 87% accuracy and 90% recall when generating structured reports from clinical images in controlled contexts, particularly when the data came from textbooks [35]. Similarly, combining images with clinical history has been seen to considerably improve the model’s diagnostic ability, achieving accuracy rates of 73.3% for OSCC and 93.3% for OL [36]. However, performance decreases significantly when isolated images are assessed, in line with our own findings.

The results obtained for OL and OLP in our study reinforce this limitation; sensitivity was 60% for OL and 25% for OLP. This restricts the usefulness of the model for ruling out potentially malignant lesions when the response is negative. These data highlight that sensitivity is the key parameter to evaluate in a clinical setting, since false negatives can delay diagnosis and compromise patient outcomes.

The discrepancy between specificity and sensitivity observed here is consistent with what has been seen in the broader field of multimodal diagnosis [37]. Although models such as GPT-4V show promise in identifying anatomical structures, their performance in specific clinical tasks is inconsistent [38]. These limitations may be due to a lack of training specific to oral pathology, variability in lesion presentation, or the use of quite a simple diagnostic prompt [39].

These limitations may be due to a lack of specific training in oral pathology, variability in lesion presentation, or the use of a remarkably simple diagnostic prompt.

The study intentionally employed a basic formulation to explore the model’s performance under minimal conditions, but it is important to acknowledge that prompt engineering can substantially influence results. Alternative prompts, such as those asking the model to describe visual features or to propose a differential diagnosis, might yield different or superior outcomes. This stands for a natural pathway for future research, as prior work has shown notable performance improvements when structured or contextual prompts are applied [4,36,39].

It is also important to clarify that the initial selection of images by general dentists did not introduce a systematic bias in the dataset. Their role was limited to identifying lesions they considered suspicious, without knowing whether these were truly positive or negative cases. The reference standard was established independently by an oral pathology specialist, with clinicopathological correlation and histopathological confirmation when available. Therefore, the validity measures reported in this study reflect comparisons exclusively against the histological gold standard, and not against the preliminary assessments made by the general dentists. This design ensured that the evaluation of ChatGPT’s performance was unbiased and aligned with the diagnostic reality.

Nevertheless, recent results suggest that ChatGPT-4o can achieve notable levels of accuracy when provided with clinically structured input. In a study using clinical images, the model achieved 58.2% diagnostic accuracy. When its proposed diagnostic tests and treatments were correct, they were accurate in 90.7% and 95.8% of cases, respectively. Furthermore, substantial to nearly perfect agreement was observed in repeated responses, indicating its potential for consistency under controlled conditions [40].

Recent studies [39] also point out that, despite the immense potential of multimodal models to integrate text, images, and audio, they are not widely used in dentistry due to challenges such as poor data quality, model bias and a lack of clinical datasets specific to dentistry.

Other studies confirm this trend. For example, Pradhan [41] reported that, although ChatGPT-4o outperforms other models when it comes to identifying potentially malignant lesions from clinical texts and images, it still lags behind human experts when it comes to visual recognition tasks. Similarly, Kaygısız and Teke [42] compared ChatGPT-4o with DeepSeek-v3 in textual clinical scenarios and observed lower diagnostic accuracy for the former (3.15 vs. 4.02 points). However, AlFarabi Ali et al. [43] demonstrated that ChatGPT can achieve performance comparable to human consultants in written clinical simulations, with 70% accuracy in differential diagnoses.

Furthermore, it has been documented that ChatGPT-4.0 can identify OL more reliably through direct visual analysis, whereas OSCC diagnosis improves when clinical history is incorporated into the prompt [36].

When placing these findings in context, it is essential to compare ChatGPT-4o with dedicated image analysis systems such as convolutional neural networks (CNNs). CNN-based tools have already shown high sensitivity and accuracy in detecting oral cancer and potentially malignant disorders from photographic images [10,12,13]. In comparison, ChatGPT-4o shows lower sensitivity and greater variability, reflecting its status as a general-purpose model not specifically trained for visual recognition of oral lesions. This contrast underscores that, while LLMs may serve as complementary aids, CNN-based approaches remain the current benchmark for automated image analysis in oral pathology.

At the same time, LLMs offer interpretability and the flexibility to integrate multimodal clinical information—qualities that CNNs alone cannot provide. Therefore, the most promising future direction may involve hybrid systems, in which CNNs handle feature extraction with high sensitivity and LLMs contribute reasoning, explanations, and integration with patient history. This combined approach could address the limitations observed in sensitivity while leveraging the strengths of LLM-based reasoning.

Despite these limitations, ChatGPT has demonstrated satisfactory performance in simulated, text-based scenarios. Several studies have shown that the model can achieve accuracy levels comparable to human professionals in clinical reasoning tasks, particularly when well-structured cases and standardized vocabulary are employed [35,43]. Nevertheless, even in these contexts, the authors agree that the model should not be considered a substitute for professional clinical judgment.

The study, despite its limitations—chief among them the small dataset (2 OSCC, 3 OL, 4 OLP)—provides an initial indication of the performance of a multimodal model that has not been specifically trained in oral pathology. The limited sample makes the estimates fragile and prevents generalization; therefore, the study’s findings should be interpreted only as preliminary observations from an exploratory pilot study. No preliminary calculation of statistical power or sample size was performed, since the main purpose of this work was exploratory. This absence, combined with the small size, further limits the robustness of the results and highlights the need for future studies to incorporate formal sample size estimation to obtain reliable measures of diagnostic validity.

Additionally, no formal statistical comparisons (e.g., between ChatGPT’s performance across the three conditions or between ChatGPT and dentists) were performed, nor were *p*-values calculated. Given the limited sample size, such analyses would lack the necessary power and could lead to misleading conclusions. This represents another important avenue for future research, where adequately powered studies should include statistical tests to determine whether observed differences are significant or merely due to chance.

Future research should also explore the impact of prompt design in more depth. The present study deliberately employed a simple formulation to test baseline performance, but structured prompts asking for stepwise reasoning, description of features, or ranking of differential diagnoses could potentially increase sensitivity. This aligns with reports from other medical domains where prompt engineering significantly improved model output [44].

Future research should include larger, more representative samples; tailored prompts for each pathology; models trained with real oral images; and comparisons with AI-based visual models. By “real oral images,” we specifically refer to prospectively collected, histologically confirmed datasets, which are essential to reduce bias and improve the reliability of training and validation processes. Based on power considerations, meticulously designed studies will likely require hundreds of well-documented and histologically confirmed cases for each pathology to generate reliable and clinically meaningful estimates.

The results obtained suggest that ChatGPT-4o could play a complementary role in OSCC screening, but it is not currently reliable enough for diagnosing OL or OLP. These findings reinforce the need for specific multimodal training, improved clinical inputs, and rigorous validation before considering its implementation in real healthcare settings.

## 5. Conclusions

ChatGPT-4o demonstrated high specificity and acceptable overall performance in screening for squamous cell carcinoma, suggesting its potential usefulness as a complementary clinical tool. However, its limited sensitivity in the detection of oral leukoplakia and lichen planus restricts its use as the only resource for ruling out these lesions.

These results must be understood as preliminary, given the pilot nature of the study and its small sample size. They confirm the need for specific training involving real oral images, as well as prompts tailored to each pathology. Future adequately powered studies, with larger and prospectively collected datasets, are essential before considering clinical implementation.

## Figures and Tables

**Table 1:** Diagnoses based on images selected by general dentists who suspected the following conditions: oral squamous cell carcinoma (OSCC), oral leukoplakia (OL) and oral lichen planus (OLP).

Condition	Reference Diagnosis	ChatGPT Diagnosis: Present	ChatGPT Diagnosis: Absent
OSCC ^A^	Present	39	21
	Absent	6	204
	Total	45	225
OL ^B^	Present	54	36
	Absent	0	30
	Total	54	66
OLP ^C^	Present	30	90
	Absent	7	173
	Total	37	263

^A^: n = 270 diagnoses, 9 images × 30 repetitions; ^B^: n = 120 diagnoses, 4 images × 30 repetitions; ^C^: n = 300 diagnoses, 10 images × 30 repetitions.

**Table 2:** Diagnostic performance metrics for ChatGPT-4o when used to detect oral squamous cell carcinoma (OSCC), oral leukoplakia (OL), and oral lichen planus (OLP).

	OSCC	95% CI		OL	95% CI		OLP	95% CI	
Sensitivity	65.00%	51.60%	76.87%	60.00%	49.13%	70.19%	25.00%	17.55%	33.73%
Specificity	97.14%	93.89%	98.94%	100.00%	88.43%	100.00%	96.11%	92.15%	98.42%
Positive predictive value	86.67%	73.21%	94.95%	100.00%	93.40%	100.00%	81.08%	64.84%	92.04%
Negative predictive value	90.67%	86.09%	94.13%	45.45%	33.14%	58.19%	65.78%	59.70%	71.50%
False positive rate	2.86%	1.06%	6.11%	0.00%	0.00%	11.57%	3.89%	1.58%	7.85%
False negative rate	35.00%	23.13%	48.40%	40.00%	29.81%	50.87%	75.00%	66.27%	82.45%
Correctly classified	90.00%	85.78%	93.31%	70.00%	60.96%	78.02%	67.67%	62.05%	72.93%
ROC area	81.07%	74.88%	87.26	80.00%	74.91%	85.09%	60.56%	56.42%	64.70%

## Data Availability

The data that support the findings of this study are available from the corresponding author upon reasonable request.

## Abbreviations

The following abbreviations are used in this manuscript:

OSCC	oral squamous cell carcinoma
OL	oral leukoplakia
OLP	oral lichen planus
AI	artificial intelligence
LLMs	large language models
OPMDs	potentially malignant disorders
PPV	positive predictive value
NPV	negative predictive value
AUC	area under the ROC curve
